# Volatile and Cuticular Chemical Profiling and Antennal Dose–Responses in *Leptoglossus chilensis* (Hemiptera: Coreidae)

**DOI:** 10.3390/insects17090923

**Published:** 2026-09-03

**Authors:** Ricardo Ceballos, Juan P. Alveal, Carla Alveal, Natalí Fernández

**Affiliations:** Laboratory of Insect Chemical Ecology, Instituto de Investigaciones Agropecuarias, INIA. Av. Vicente Méndez 515, Chillán 3800062, Chile

**Keywords:** Coreidae, electroantennography, dose–response, Bayesian hierarchical model, semiochemicals

## Abstract

*Leptoglossus chilensis* is a native South American coreid bug of economic and quarantine relevance whose chemical ecology remains undescribed. In this exploratory study, we characterised adult volatile and cuticular chemistry using dynamic headspace sampling (DHS), cuticular wash (CW), solid-phase microextraction (SPME), supercritical fluid extraction (SFE), and quantified electroantennographic (EAG) dose–responses in males and females in relation to a panel of eight literature-informed compounds (hexanal, hexyl acetate, hexyl formate, β-caryophyllene, (*E*)-β-farnesene, ocimene, benzyl alcohol and 2-phenylethanol). EAG responses were evaluated at six concentrations using a Bayesian hierarchical four-parameter Hill model. Across the four methods, 128 compounds were detected, of which 69 met a ≥20% detection-frequency threshold and were retained as the representative profile. Of these, hexanal, hexyl acetate, hexyl formate, and β-caryophyllene were detected in *L. chilensis* extracts, the latter only in male DHS collections; this compound is more likely to be a host plant volatile than an insect-produced signal. All eight compounds elicited concentration-dependent antennal responses in both sexes. Among these four compounds, β-caryophyllene yielded the lowest fitted EC_50_ in both females (9 µg mL^−1^ [90% CrI: 5–16]) and males (9 µg mL^−1^ [90% CrI: 5–15]), although curves remained unsaturated; because delivered antennal dose was not measured and volatility differs substantially among the tested compounds, this cannot be interpreted as evidence of greater antennal sensitivity to β-caryophyllene specifically, and cross-compound EC_50_ comparisons throughout this study are restricted to this caveat. All four Hill parameters were identifiable in both sexes for benzyl alcohol, (*E*)-β-farnesene, hexyl formate, and 2-phenylethanol. These results provide the first chemical and peripheral olfactory reference for *L. chilensis* and identify targets for behavioural and field evaluation.

## Simple Summary

*Leptoglossus chilensis* is a native Chilean insect that damages fruit and nut crops and is a frequent reason fresh fruit shipments from Chile are rejected at import. Farmers currently rely on insecticide sprays because there is no lure or monitoring tool, as the chemical signals this insect uses to communicate were not previously characterised. We studied adult males and females using several methods to collect the compounds present on their body surfaces and in the air around them, and we measured how their antennae responded to eight odours already known from related pest species elsewhere. We detected more than 100 compounds, several matching those used by related species, and found that a compound initially suspected to be a male insect signal is more likely to originate from the host plant, an important distinction to resolve in future work. All eight odours triggered a response from the antennae, showing that this insect can detect them, even though their biological meaning remains untested. These results provide the first chemical and sensory reference for this species and identify candidate compounds to guide future odour-based monitoring tools, which could reduce insecticide use and help prevent costly quarantine rejections of Chilean fruit exports.

## 1. Introduction

The genus *Leptoglossus* (Hemiptera: Coreidae) comprises more than 60 predominantly phytophagous species, several of which are significant pests in agriculture and forestry [1,2,3]. Commonly known as leaf-footed bugs, these insects feed on various fruit, vegetable, and seed crops using piercing–sucking stylets that damage developing seeds and fruits, frequently causing malformations, pod abortion, and decreased seed quality, and can also promote infection by pathogenic microorganisms [4,5]. Chemical communication in *Leptoglossus* is mediated by a complex repertoire of semiochemicals; nymphs release deterrent compounds from their dorsal abdominal glands, while adults rely on metathoracic scent glands to emit defensive volatiles, alarm pheromones, and aggregation signals [1,6]. In several species, adult males additionally produce sex and aggregation pheromones that attract conspecifics, including a male-specific aggregation pheromone in *L. occidentalis* [7] and sex pheromone components in *L. zonatus* [8] and *L. clypealis* [9].

*Leptoglossus chilensis* (Spinola) is a native South American leaf-footed bug recorded in Argentina, Brazil, Chile, Paraguay, and Uruguay [10]. In Chile, it is associated with a wide range of native hosts, including *Lithraea caustica*, *Peumus boldus*, and *Quillaja saponaria* [11]. The species has long been recorded as a pest of economically important crops, including walnuts and stone fruits, and since the early 1950s has been the most frequently detected cause of quarantine rejections in fresh fruit exports from Chile to the United States [12,13]. It is also listed in the U.S. Regulated Plant Pest Table maintained by USDA–APHIS Plant Protection and Quarantine [14]. More recently, the rapid expansion of hazelnut orchards in central and southern Chile has been accompanied by increasing field observations of *L. chilensis* feeding on developing nuts during mid-kernel fill, leading to fruit malformation, seed abortion, and loss of kernel quality. Current management strategies rely predominantly on visual surveillance coupled with insecticide applications, with the absence of semiochemical-based monitoring tools reflecting the complete lack of chemical ecology knowledge for this species. Despite its economic and quarantine relevance, the chemical ecology of *L. chilensis* remains entirely uncharacterised; no pheromone, defensive secretion, or semiochemical mediating intraspecific or host-finding chemical communication has been identified for this species. This knowledge gap has become increasingly consequential given the recent changes in the *Leptoglossus* fauna of Chile. The invasive *L. occidentalis*, associated with conifers, was first detected in Valparaíso in 2017 and has since expanded rapidly from Atacama to Los Lagos [15], while *L. crassicornis*, a species associated with *Cactaceae* and distributed from Honduras to central Argentina, has also been recorded for the first time in the Valparaíso and Metropolitan regions [16]. Chile therefore harbours four co-occurring *Leptoglossus* species, native *L. chilensis* and *L. impictus*, and recently introduced *L. occidentalis* and *L. crassicornis* [15,16]. Of these, only *L. occidentalis* has partially characterised semiochemicals, including aggregation pheromone components and male-specific sesquiterpene leptotriene. The latter was also identified in *L. zonatus*, a congener not recorded in Chile whose alarm pheromone constituents are among the best-characterised in the genus [8,17,18].

A necessary first step toward understanding the chemical ecology of *L. chilensis* is to characterise the volatile and cuticular compounds associated with adults and to determine whether the peripheral olfactory system detects selected compounds of potential semiochemical relevance. The documented conservation of semiochemicals across *Leptoglossus* species supports this approach; C_6_ aliphatic alarm components are shared across developmental stages in *L. zonatus* and elicit responses even across family [17], while leptotriene, a male-specific sesquiterpene, is independently produced by both *L. zonatus* and *L. occidentalis* [18]. This recurrent pattern of shared compounds within the genus provides a rational basis for extending the EAG stimulus panel to include compounds reported from congeners, while recognising that their behavioural relevance in *L. chilensis* remains untested. Establishing peripheral antennal sensitivity to this panel provides a basis for subsequent GC-EAD and behavioural evaluation. The electroantennogram (EAG) provides a robust and efficient method for this purpose. As a summed receptor potential recorded from the antenna, it reflects the aggregate response of olfactory receptor neurons to a given stimulus [19], providing a direct, tractable measure of peripheral sensitivity before more demanding behavioural bioassays. As an exploratory characterisation of a previously undescribed chemical ecology system, our objectives were to characterise the volatile and cuticular chemical profiles of adult *L. chilensis* using four complementary, non-redundant extraction methods, and to quantify peripheral antennal sensitivity to a literature-informed panel of compounds representing semiochemical classes reported in *Leptoglossus*. The latter approach evaluates whether *L. chilensis* antennae respond to compound classes reported from congeners. Antennal responses to congener-derived compounds would indicate peripheral antennal responsiveness to compound classes reported elsewhere in the genus. Given reported overlap in semiochemical classes within *Leptoglossus*, we predicted that *L. chilensis* would be associated with C_6_ aldehydes and esters characteristic of coreid defensive chemistry, and that its antennae would respond to selected congener-derived compounds. We further expected sex-associated differences in chemical profiles, given sex-biased glandular chemistry reported in congeners.

Here, we characterise adult-associated volatile, cuticular, and extractable chemical profiles of *L. chilensis* using four complementary extraction methods and quantify peripheral antennal responses in males and females to eight selected volatile compounds at six nominal concentrations (0.1–10,000 µg mL^−1^). EAG dose–response curves were fitted using a four-parameter Hill model within a Bayesian hierarchical framework implemented in brms [20], and posterior distributions for *E*_0_, *E*_max_, EC_50_, and *n* were compared between sexes for each compound.

## 2. Materials and Methods

Insects. Adults *Leptoglossus chilensis* of both sexes were collected manually from aggregations on *Datura stramonium* L. (Solanaceae) during morning collection sessions (09:00–12:00 h) at the start of each week, at the Universidad de Concepción campus Chillán, Ñuble Region, Chile (36.599° S, 72.084° W), between November 2025 and January 2026. Only adults were collected, and were distinguished from nymphs by external morphology, consistent with standard practice for field-collected Heteroptera in chemical ecology studies. Species identification was confirmed by the authors with the support of using Brailovsky’s [2] illustrated key. Insects were transported to the laboratory, where the sex was determined by the external morphology of the ninth abdominal segment and individuals were separated by sex, and then kept separately by sex in mesh cages (40 × 40 × 50 cm) containing a potted *D. stramonium* plant regularly watered to maintain turgor. Sucrose solution (10% *w*/*v*) was provided ad libitum via cotton wicks. Insects were maintained at 23–26 °C and 50–60% relative humidity under natural ambient photoperiod at the study latitude (36.6° S), and used within the same week of collection. Adult voucher specimens of both sexes were deposited at the Museo de Zoología, Universidad de Concepción (MZUC-UCCC), Concepción, Chile, under accession number MZUC-UCCC 49023.

Dynamic headspace sampling (DHS). Chemical (DHS, CW, SFE, and SPME) collection sessions began at 14:00 h, fixing chemical sampling to a consistent afternoon/evening/overnight window across all replicates and methods; within-day rhythmicity of volatile emission itself was not characterised (see Section 4). The volatiles emitted were collected from 10 replicate groups of 30 adult *L. chilensis* per sex. Insects were placed in 200 mL glass chambers under a positive–negative air pressure system with an inflow rate of 0.6 L min^−1^ and an outflow rate of 0.25 L min^−1^; chambers were maintained under slight positive pressure to minimise ambient air contamination. Inflow air was purified through a 30 g activated charcoal filter. The volatiles were collected over 18 h onto Porapak Q traps (300 mg, 80–100 mesh), previously rinsed with 1 mL anhydrous diethyl ether and thermally conditioned at 220 °C for 2 h under nitrogen flow (50 mL min^−1^). After collection, volatiles were eluted from the traps with 1 mL chromatographic-grade hexane containing 10 ng µL^−1^ of 1,2,3,4-tetrahydronaphthalene (tetralin) as internal standard. To evaluate potential plant contributions to the detected headspace profile, additional DHS collections were performed from *D. stramonium* leaves under conditions matching the insect protocol, except that collection time was extended to 24 h (vs. 18 h for insects) to ensure sufficient volatile accumulation from plant material. Ten replicate leaf samples (30 g fresh leaf tissue each) were collected from vegetative-stage plants without flowers or fruit, matching the foliage available in insect rearing cages. No insects were present during these collections.

Cuticular wash. Cuticular compounds were independently extracted from 30 adult *L. chilensis* of each sex; however, a male sample was lost during processing, resulting in 29 valid male extractions. Prior to extraction, the individuals were deprived of food for 24 h to minimise contamination from plant-derived surface deposits and faeces, then frozen at −20 °C for 1 h. Each insect was placed in 1 mL of chromatographic-grade hexane in an amber vial for 30 s [21] with gentle manual shaking. The resulting extract was passed through a 0.22 µm Millipore membrane filter, transferred to a clean vial, and concentrated to dryness under a gentle nitrogen flow (50 mL min^−1^). The residues were then redissolved in 100 µL of chromatographic-grade hexane containing tetralin (10 ng µL^−1^) as an internal standard.

Supercritical fluid extraction (SFE). The semi-volatile compounds were extracted from 10 replicate groups of 20 adult *L. chilensis* per sex. Prior to extraction, insects were frozen at −20 °C for 3 h. The extraction chamber (Nomadic Extractor, SFE Process, Tomblaine, France) was pre-conditioned at 10 ± 1 °C for 1 h before loading. The system was pressurised with liquid CO_2_ to 40 bar, after which temperature was progressively increased to 90 ± 1 °C, reaching a final pressure of 90 bar; these conditions were maintained for 30 min. Extracts were captured on Porapak Q traps (300 mg, 80–100 mesh) conditioned as described above and eluted with 1 mL chromatographic-grade hexane containing tetralin (10 ng µL^−1^) as an internal standard.

Solid-phase microextraction (SPME). The volatile compounds were extracted from 10 replicate groups of 20 adult *L. chilensis* per sex. To minimise contamination from food residues and excreta, insects were starved for 30 min prior to analysis and then introduced into 200 mL glass chambers maintained at 23 ± 1 °C for 15 min for acclimation. Volatiles were collected by exposing a solid-phase microextraction fibre (50/30 µm DVB/CAR/PDMS, Stableflex, 24 Ga, manual holder; Supelco, Bellefonte, PA, USA) to the chamber headspace for 15 min. Adsorbed compounds were thermally desorbed directly in the GC injector for 2 min. The fibre was conditioned before use following the manufacturer’s specified time and temperature; no separate carryover-blank runs beyond this manufacturer-specified conditioning were performed. No internal standard was used; therefore, compound abundances are reported as relative peak area (%) and are not directly comparable with quantification from other extraction methods.

GC-MS analysis. The identification and semi-quantification of compounds followed identical instrumental conditions for all four extraction methods. Samples (1.0 µL) were injected in splitless mode into a gas chromatograph coupled to a mass spectrometer (QP2010 Ultra, Shimadzu, Kyoto, Japan) equipped with an InertCap 5MS capillary column (5% diphenyl–95% dimethylpolysiloxane; 60 m × 0.25 mm i.d. × 0.25 µm film thickness; GL Sciences, Tokyo, Japan). The injector temperature was set to 250 °C. Helium was used as the carrier gas at a constant column flow of 1.0 mL min^−1^. The oven temperature program was 40 °C for 1 min, ramped at 10 °C min^−1^ to 280 °C, and held for 10 min, with a total run time of 35 min.

Linear retention indices [22] were calculated using a homologous series of n-alkanes (C_8_–C_28_) analysed under identical chromatographic conditions. Because the alkane calibration run and the chromatographic method used here do not resolve standards beyond approximately C_28_–C_30_, linear retention indices could not be computed for compounds eluting outside this window—either beyond the heaviest standard (n-C_28_) or before the lightest standard (n-C_8_); for these compounds, identification relies on mass-spectral library matching alone (similarity ≥ 90%), without retention-index corroboration, and is reported as such in Table S2. For SPME, per-replicate raw retention times were not retained in the archived dataset, so indices for this technique are reported on the original logarithmic (Kovats) scale rather than recalculated as linear indices. For each of the four extraction methods (DHS, CW, SFE, and SPME), procedural blanks (solvent only, no insect) were run through the full corresponding sampling workflow. Peaks detected in these blank collections were excluded from sample chromatograms at matching retention times, to remove system- or background-derived signals from the representative chemical profile. Most compounds were tentatively identified by comparison of experimental retention indices with published values and by mass spectral matching against the NIST/Wiley library using a similarity threshold ≥ 90%. Identifications were flagged as tentative when the linear retention index deviated from the published value by more than 15 index units (absolute) and identity was not confirmed by co-injection with an authentic standard. Full identification data (similarity, experimental and library retention indices, deviation) for all compounds across the four extraction methods are provided in Table S2 in Zenodo (https://doi.org/10.5281/zenodo.22136329). Tentative identifications were evaluated for plausibility of biological origin. 1,3-Dimethylbenzene was considered a likely environmental or laboratory-derived contaminant, given its common occurrence as a solvent and plasticiser component and the absence of precedent for this compound in Coreidae or Heteroptera chemical ecology. 2-Heptadecyloxirane, an epoxide of a long-chain alkene, was considered a possible autoxidation artefact of a cuticular hydrocarbon precursor arising during sample processing, rather than a native compound. 2,4-Dimethylhexane is reported as a tentative identification without sufficient evidence to assign either contaminant or insect-derived origin. DHS and SFE extracts were semi-quantified relative to the internal standard tetralin (10 ng µL^−1^) and normalised by the number of insects per chamber; abundance is expressed as ng insect^−1^. CW extracts were similarly quantified relative to the internal standard and expressed as ng insect^−1^ based on individual extractions. SPME compound abundances are reported as relative peak area (%) because no internal standard was used. As this study constitutes an exploratory characterisation of a previously undescribed chemical ecology system, insect numbers per replicate were set according to the throughput and material requirements of each extraction technique, individual processing for CW versus pooled chamber sampling for DHS, SFE, and SPME, rather than through a priori power analysis, and therefore differ across methods.

For descriptive summaries of the representative chemical profile, compounds were retained when detected in ≥20% of biological replicates in at least one sex within a given extraction method. This threshold was used to reduce the influence of sporadic low-frequency detections and trace background signals. The complete GC-MS dataset, including low-frequency detections below this threshold, is available in Zenodo (https://doi.org/10.5281/zenodo.22136329). Compounds selected for EAG bioassays were reported independently of this frequency filter because the EAG panel was defined a priori from representative semiochemical classes reported in *Leptoglossus*.

Sex differences in compound detection frequency, described narratively below, were additionally tested formally. For every compound within each extraction method, a two-sided Fisher’s exact test compared female vs. male detection counts among that method’s biological replicates (CW: nF = 30, nM = 29, individual insects; DHS, SFE, SPME: nF = nM = 10, pooled chambers of 20–30 insects per replicate, the biological replicate already defined above for these methods). The false discovery rate was controlled across all 210 compound×method comparisons using the Benjamini–Hochberg procedure. Because DHS/SFE/SPME replicates are pooled chambers, these tests assess between-chamber rather than between-individual differences for those three methods; only the CW tests assess between-individual differences directly.

Stimulus compounds. Eight compounds were selected a priori as a literature-informed, standards-constrained EAG panel representing chemical classes implicated in *Leptoglossus* chemical ecology. Selection prioritised compounds or compound classes reported in *Leptoglossus* semiochemical studies and available as authentic standards. Detection of these compounds in *L. chilensis* extracts was evaluated independently. The panel included C_6_ aldehyde and ester compounds associated with alarm or defensive chemistry (hexanal, hexyl acetate, hexyl formate); sesquiterpene hydrocarbons associated with male emissions or aggregation signals (β-caryophyllene, (*E*)-β-farnesene); aromatic alcohols reported from male glandular secretions (benzyl alcohol, 2-phenylethanol); and a monoterpene previously reported as electrophysiologically or behaviourally active in *Leptoglossus* species (ocimene) [8,18,23,24].

Analytical standards were obtained from Sigma-Aldrich (hexanal 98%, (*E*)-β-farnesene 90%, β-caryophyllene 98%, hexyl acetate 99%, hexyl formate 97%, and ocimene (mixture of isomers, ≥90%)), Supelco (2-phenylethanol 99.3%), and CPA Chem (benzyl alcohol 99.7%), and dissolved in chromatographic-grade hexane. Serial dilutions yielded six concentrations spanning five orders of magnitude (0.1, 1, 10, 100, 1000, and 10,000 µg mL^−1^). For stimulus delivery, 25 µL of each solution were deposited onto a filter paper strip (1 × 5 cm) inserted into a glass Pasteur pipette cartridge, yielding nominal stimulus loads ranging from 2.5 ng to 250 µg per cartridge. Cartridges were single-use, discarded after one presentation series, and not reused across antennae or replicates; the interval between loading the filter-paper strip and the first stimulus presentation was held constant at ~60 s, of which the first ~30 s allowed evaporation of excess solvent. Here, “dose–response” is used operationally to refer to EAG responses measured across the nominal concentrations of stimulus solutions applied to filter-paper cartridges; the actual amount of compound released from the cartridge and delivered to the antenna was not quantified. Hexane (25 µL) served as solvent control. Solutions were kept at −20 °C and used within the same week in which they were prepared. Dose–response modeling used the nominal solution concentration (µg mL^−1^) as the predictor variable. Because the actual rate of compound release from filter-paper cartridges was not quantified, EC_50_ estimates represent the solution concentration yielding half-maximal antennal response under these delivery conditions, not the absolute amount of compound reaching the antenna.

Antenna preparation and stimulus delivery. A single antenna was excised from each individual at random (left or right), with one preparation per individual to avoid pseudoreplication. The excised antenna was immediately mounted between two glass capillary electrodes filled with Beadle–Ephrussi Ringer solution [25] (NaCl 7.5 g L^−1^, KCl 0.35 g L^−1^, CaCl_2_·2H_2_O 0.29 g L^−1^, and polyvinylpyrrolidone 5 g L^−1^ [0.5% *w*/*v*] as a colloid/osmotic-support additive not in the base published recipe; unbuffered, pH not measured or adjusted), with the antennal base connected to the reference electrode and the apex (distiflagellum) to the recording electrode; electrical contact with the preamplifier was maintained via gold wire (0.4 mm). Electrode positioning was controlled with micromanipulators (MP-15, Syntech, Kirchzarten, Germany). EAG recordings were obtained using a Syntech system (Kirchzarten, Germany) comprising an EAG COMBI probe, CS-55 stimulus controller, and IDAC-4 data acquisition controller; signals were recorded with GC-EAD software v. 1.3.1. Preparations were accepted when stable baseline activity and repeatable responses to air pulses were observed prior to stimulation. Recordings typically commenced within 2–3 min of excision.

Stimuli were delivered as 1 s pulses of compound-laden air (250 mL min^−1^) directed onto the antenna preparation via a glass Pasteur pipette cartridge loaded with 25 µL of stimulus solution on a filter paper strip, integrated into a continuous humidified airstream at ambient temperature. Each preparation was exposed sequentially to all concentrations of a single compound in ascending order (0.1 to 10,000 µg mL^−1^). The inter-stimulus interval was 60 s, during which two 1 s air pulses at 30 s intervals verified antenna responsiveness and facilitated receptor clearance. Ascending concentration order was used to prioritise detection of low-threshold responses, while recognising that this design does not fully separate concentration effects from presentation-order effects [26,27]. A linalool (97%, Sigma-Aldrich) presentation (1000 µg mL^−1^) was included before each replicate series as an internal physiological reference; an additional presentation (10,000 µg mL^−1^) at the beginning and end of each session served as a fatigue control. Responses were normalised relative to the nearest linalool reference to account for signal drift. EAG amplitude was defined as the absolute voltage deflection from baseline to peak response (mV). Data quality control and drift correction procedures are described in File S1.

Statistical analysis. EAG amplitude was modelled as a nonlinear function of nominal stimulus concentration using a four-parameter Hill dose–response equation:

$$\mu \left(d\right)=E_{0}+\left(E_{max}-E_{0}\right)\frac{d^{n}}{{EC}_{50}^{n}+d^{n}}$$

where *d* is the stimulus concentration (µg mL^−1^), *E*_0_ is the lower asymptote or baseline amplitude, *E*_max_ is the upper asymptote or maximum response, EC_50_ is the concentration eliciting a half-maximal response, and *n* is the Hill slope coefficient.

All four parameters were estimated on the log scale to enforce positivity and back-transformed for reporting. Models were fitted in a Bayesian hierarchical framework using brms v.2.23.0 [20] and CmdStan v.2.38.0. A Student-t error distribution was used to reduce the influence of occasional large EAG deflections. For each compound, two random-effects structures were compared: a baseline model (G1), with a random intercept on *E*_0_, and an extended model (G2), with random intercepts on both *E*_0_ and *E*_max_. These structures allowed between-antenna variation in baseline response and, where supported, in maximum response.

Model selection was performed independently for each compound using leave-one-out cross-validation (LOO-CV; [28]). Expected log pointwise predictive density (*elpd*) and the standard error of the *elpd* difference were computed for the G1 and G2 specifications. Δ*elpd* was calculated as elpd_G1–elpd_G2; therefore, negative values indicate higher expected predictive performance of G2. To avoid selecting the more complex random-effects structure when predictive improvement was weak relative to its uncertainty, G2 was retained only when Δ*elpd*/SE Δ < −2; otherwise, the simpler G1 model was used for subsequent inference. Observation-level LOO-CV was computed first, at the level of individual observations rather than antenna groups; because observations within each antenna are not fully exchangeable with those from other antennas, absolute elpd estimates from this comparison may be optimistic. To address this, model selection was repeated using 10-fold cross-validation grouped by antenna (whole antennas held out of each training fold, via brms::kfold()), which does not share this limitation. This grouped-antenna comparison, rather than the observation-level comparison, was adopted as the primary model-selection criterion for all reported results. Weakly informative priors were specified on the log scale as Normal(log(0.35), 0.4) for *E*_0_, Normal(log(1.0), 0.5) for *E*_max_, Normal(log(100), 1.5) for EC_50_, and Normal(log(2), 0.5) for *n*. Exponential(2) priors were assigned to between-antenna standard deviations and residual SD, and a Gamma(2, 0.1) prior was assigned to the Student-t degrees-of-freedom parameter. Because some dose–response curves were expected to remain incomplete within the tested concentration range, parameter identifiability was assessed from the posterior distributions. Curves were flagged as NE† when the upper asymptote was not constrained by the data, as NI when EC_50_ was not identifiable from the posterior distribution, and as § when EC_50_ was model-estimable within the tested concentration range but *E*_max_ remained unconstrained. In the latter case, EC_50_ should be interpreted as a model-implied inflection parameter rather than as a fully empirical half-maximal concentration. Prior sensitivity was evaluated by varying the EC_50_ prior SD across three specifications (1.0, 1.5, and 2.0); estimates were considered prior-sensitive when the ratio of posterior medians between the loosest and tightest specifications exceeded 2.0 (‡). Full details are provided in File S1. All analyses were conducted in R v.4.5.3 [29].

## 3. Results

Chemical characterisation: Compounds co-eluting with peaks detected in blank collections at matching retention times were excluded prior to compound retention filtering. Three compounds warranted cautious interpretation regarding their inclusion in the representative profile: 1,3-dimethylbenzene, likely a laboratory or environmental contaminant; 2-heptadecyloxirane, a probable autoxidation artefact of a cuticular hydrocarbon; and 2,4-dimethylhexane, a tentative identification of uncertain origin. Across the four extraction techniques, 128 unique compounds were detected. After applying a ≥20% detection-frequency threshold for at least one sex within each method, 69 unique compounds were retained as the representative chemical profile of *L. chilensis*. Because several retained compounds occurred in more than one method, these 69 compounds represented 114 method–compound occurrences, with 25 in CW, 40 in DHS, 16 in SPME, and 33 in SFE. The retained profiles were largely complementary, with 40 compounds exclusive to a single method, 16 were retained in two methods, 10 in three methods, and three in all four methods. Method-exclusive compounds were most numerous in DHS (13), followed by CW (11), SFE (10), and SPME (6). The three compounds retained across all four methods were hexanal, tetradecane, and hexanoic acid; the 10 retained in three methods were 1,3-dimethylbenzene, 2,4-dimethylhexane, 2-butyl-2-octenal, an unidentified long-chain hydrocarbon (2-methyloctacosane, CW/DHS), butyl acetate, heptadecane, heptyl acetate, an unidentified long-chain hydrocarbon (CW), hexyl acetate, and hexyl formate. CW was dominated by linear and methyl-branched alkanes, whereas DHS, SPME, and SFE recovered complementary volatile and semi-volatile fractions. Full compound lists, including low-frequency detections excluded from the representative profile, are provided in Zenodo (https://doi.org/10.5281/zenodo.22136329).

Detection frequencies varied descriptively between sexes in both cuticular and headspace profiles (Table 1). Of the 210 compound × method comparisons tested by Fisher’s exact test, 12 remained significant after Benjamini–Hochberg correction (q < 0.05; full results in the accompanying Zenodo table), confirming these as sex-differential patterns rather than descriptive impressions: male-biased detection of tetradecane, undecane, an unidentified long-chain hydrocarbon (2-methyloctacosane, CW/DHS), 2,4-dimethylhexane, and eicosane in CW, and of hexadecane and β-caryophyllene in DHS and 4-methoxyphenol in SFE; and female-biased detection of 2-heptadecyloxirane, hexadecane, and α-pinene in CW, and of an SPME ester compound detected in all female but no male replicates. Other sex-differential patterns visible in Table 1 did not survive multiplicity correction (e.g., hexanal’s apparent female bias in CW, q = 0.386) and should be read as descriptive, non-significant trends rather than confirmed sex differences.

Detection frequencies for CW compounds followed a bimodal distribution, with a core of high-frequency compounds and a tail of lower-frequency, more variable detections. Eleven compounds were detected in ≥50% of biological replicates of at least one sex (e.g., an unidentified long-chain hydrocarbon (CW): 96.7% of females, 89.7% of males; tetradecane: 86.2% of males; undecane: 75.9% of males; 2,4-di-tert-butylphenol: 69.0% of males), whereas compounds retained below this threshold were detected in 20–49% of replicates. Full bootstrap-resampled detection frequencies and 95% confidence intervals for all retained CW compounds are provided in Zenodo (https://doi.org/10.5281/zenodo.22136329).

Of the eight compounds selected for EAG bioassays, hexanal, hexyl acetate, hexyl formate, and β-caryophyllene were detected in *L. chilensis* extracts (Table 2), whose identities were confirmed by co-injection with authentic standards under identical chromatographic conditions. Hexanal was retained in the representative profile in all four methods and detected at 100% frequency in DHS, SPME, and SFE collections for both sexes. Hexyl acetate and hexyl formate were detected in all four methods but met the ≥20% threshold only in DHS, SPME, and SFE. β-caryophyllene was retained only in male DHS collections (80%) and was absent from female DHS collections and from all other extraction methods. Benzyl alcohol, 2-phenylethanol, (*E*)-β-farnesene, and ocimene were not detected in *L. chilensis* extracts under the sampling conditions used here.

Among host plant volatiles, β-caryophyllene was detected in 7 of 10 leaf DHS replicates (70%), with high spectral similarity to the library match (90–97%) and concentrations ranging from 0.69 to 10.94 ng µL^−1^ 100 g^−1^. Hexanal was not detected in any leaf sample; the only C_6_ aldehydes present in leaf headspace were the unsaturated homologs (*E*)-2-hexenal and 3-hexenal, structurally distinct from hexanal.

EAG dose–response: Model selection used 10-fold cross-validation grouped by antenna as the primary criterion (Section 2), which selected G2 (random intercepts on both log *E*_0_ and log *E*_max_) for all eight compounds tested (Δelpd/SE Δ ranging from −3.19 to −7.05 across compounds). The supplementary observation-level LOO-CV results (Table 3) agreed with this selection for five of the eight compounds but favoured the simpler G1 specification for benzyl alcohol, hexyl acetate, and ocimene (Δelpd/SE Δ = −1.52, −1.38, and −1.30, respectively); because observation-level LOO-CV can be optimistic when observations within an antenna are not fully exchangeable, we resolved this disagreement in favour of the grouped antenna criterion and report G2 for all eight compounds throughout.

All eight compounds elicited concentration-dependent EAG responses in both sexes, with posterior median estimates of the lower asymptote of the fitted response curve (*E*_0_) ranging from 0.27 to 0.50 mV across compound–sex combinations (Table 4). Dose–response curves reached an estimable upper asymptote (*E*_max_) in 14 of the 16 compound–sex combinations. Curves remained unsaturated (NE†) for β-caryophyllene in males and hexanal in females, indicating that the maximum antennal response to these stimuli was not reached within the tested concentration range in these two compound–sex combinations.

**Figure 1:**
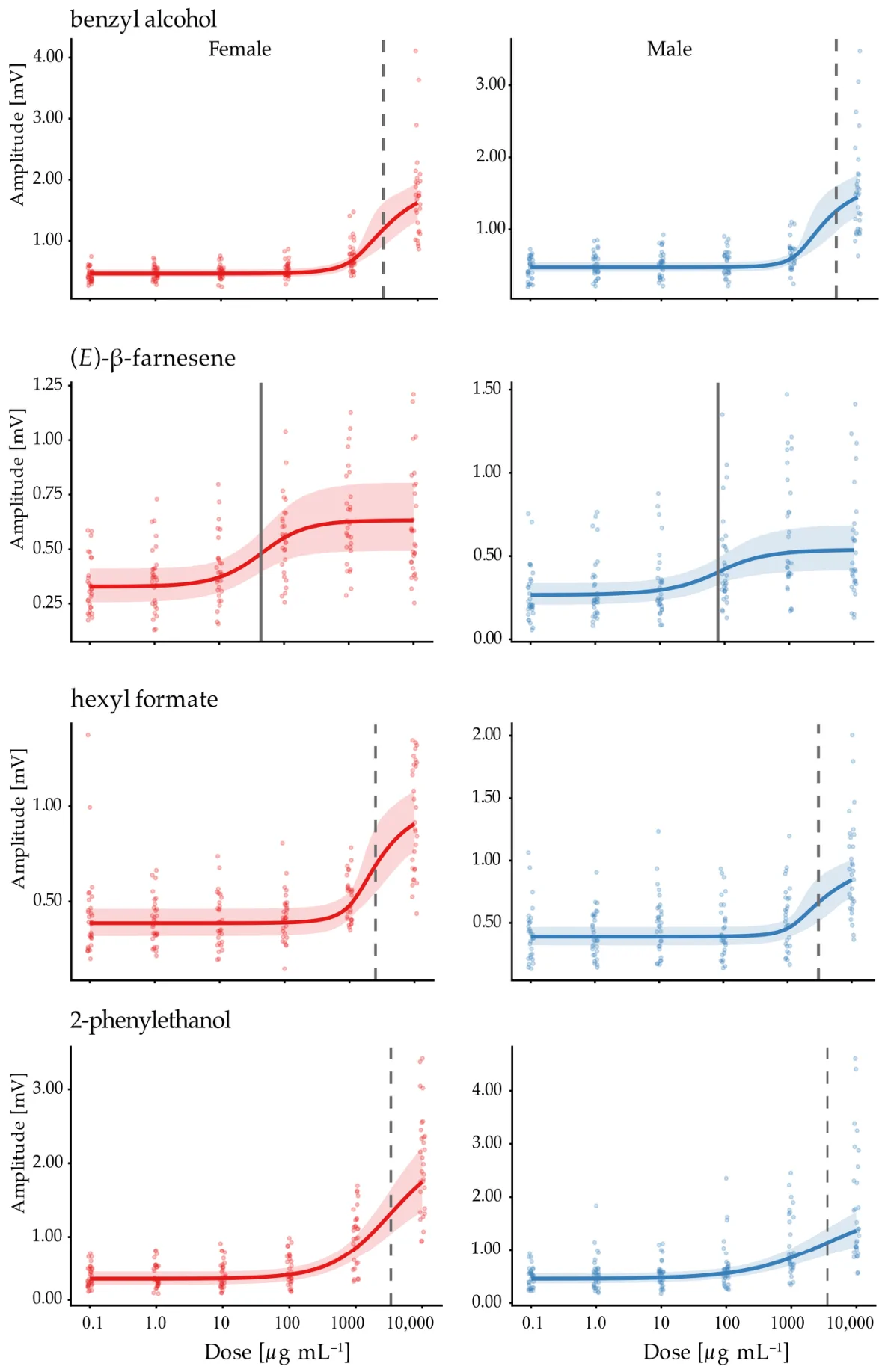
EAG dose–response curves for compounds with all four Hill parameters identifiable in both sexes. Points represent individual dose–response measurements (n = 10 antennas per sex, 6 doses per antenna), solid lines indicate posterior median fits, and shaded bands indicate 90% credible intervals. Vertical lines indicate posterior median EC_50_ estimates; solid vertical lines denote curves with an estimable upper asymptote, whereas dashed vertical lines denote unsaturated curves in which *E*_max_ was not estimable within the tested concentration range. Posterior summaries for *E*_0_, *E*_max_, EC_50_, and *n* are reported in Table 4. Full diagnostic classifications for each compound and sex, including EC_50_ identifiability, prior sensitivity flags, and random-effects structure, are provided in Table S1.

EC_50_ was identifiable in 15 of 16 compound–sex combinations (Table 4). Fitted dose–response curves are shown for the four compounds in which all four Hill parameters were identifiable in both sexes: benzyl alcohol, (*E*)-β-farnesene, hexyl formate, and 2-phenylethanol (Figure 1). Curves for the remaining compounds are provided in Figure S1. EC_50_ could not be identified (NI) for hexanal in females; this case also showed an unsaturated curve. Among compound–sex combinations with identifiable EC_50_, β-caryophyllene yielded the lowest estimates in both females (9 µg mL^−1^ [90% CrI: 5–16]) and males (9 µg mL^−1^ [5–15]); in males, where the dose–response curve remained unsaturated, this estimate is flagged (§) as reflecting the model-fitted inflection parameter rather than an empirically constrained half-maximal concentration. (*E*)-β-farnesene showed the next lowest EC_50_ values, with females (45 µg mL^−1^ [29–71]) showing lower estimates than males (68 µg mL^−1^ [41–97]), although credible intervals overlapped. The remaining identifiable EC_50_ estimates were in the high µg mL^−1^ range, hexyl acetate females (708 µg mL^−1^ [373–1632]) and males (1596 µg mL^−1^ [700–5097]), hexyl formate females (2411 µg mL^−1^ [1454–7202]) and males (3017 µg mL^−1^ [1512–12071]), benzyl alcohol females (2624 µg mL^−1^ [1473–6783]) and males (2413 µg mL^−1^ [1515–5784]), hexanal males (3598 µg mL^−1^ [1478–18243]), ocimene females (2649 µg mL^−1^ [964–9202]) and males (877 µg mL^−1^ [260–3557]), and 2-phenylethanol females (3492 µg mL^−1^ [2193–6952]) and males (2466 µg mL^−1^ [1434–6172]). Four EC_50_ estimates were flagged as prior-sensitive (‡), hexanal males, hexyl formate males, and ocimene in both sexes; these estimates should therefore be interpreted with caution.

**Figure 2:**
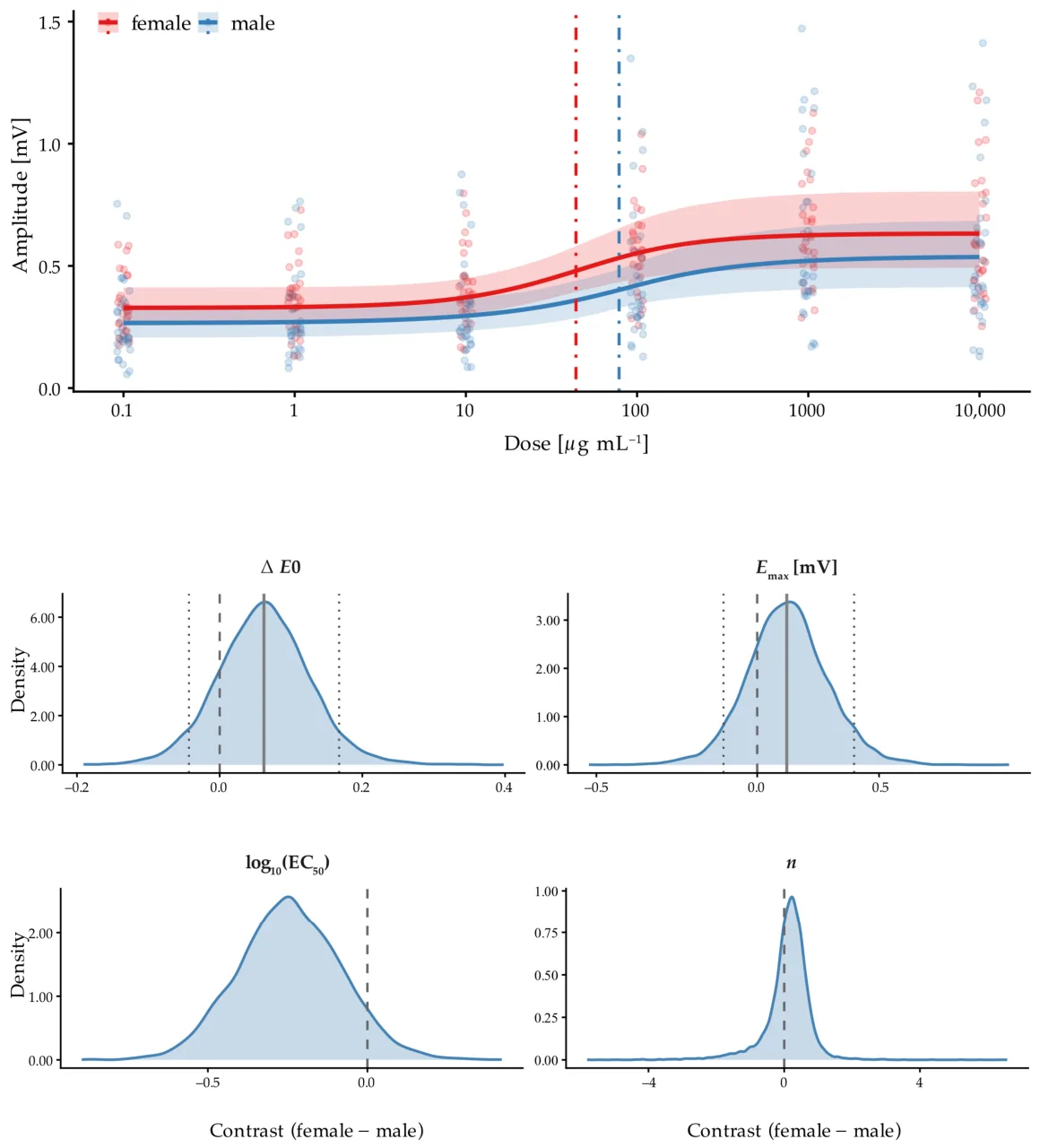
Posterior contrasts between females and males for the model parameters of (*E*)-β-farnesene. The upper panel shows fitted dose–response curves by sex. The lower panels show posterior distributions of female–male contrasts for *E*_0_, *E*_max_, log_10_(EC_50_), and *n*. Dashed lines indicate posterior medians and dotted lines indicate 90% credible intervals.

Sex-related differences in EAG parameters were modest overall. Posterior estimates of *E*_0_ largely overlapped between sexes for all compounds. Where *E*_max_ was estimable in both sexes, females tended to show higher posterior median upper asymptotes than males, most notably for 2-phenylethanol (females: 2.35 mV [1.85–3.07]; males: 1.90 mV [1.52–2.47]) and benzyl alcohol (females: 2.04 mV [1.61–2.83]; males: 1.58 mV [1.28–2.07]), although credible intervals overlapped. The Hill slope parameter (*n*) was generally similar between sexes within compounds, with values above 1 for most compound–sex combinations; hexyl acetate and ocimene were exceptions, with *n* < 1 in both sexes (Table 4). Sex-related contrasts for all four Hill parameters were computed for each compound where both sexes yielded identifiable estimates; full posterior contrast summaries are available in Zenodo (https://doi.org/10.5281/zenodo.22136329). Among compounds with estimable EC50 in both sexes, (*E*)-β-farnesene showed directional evidence of higher female antennal responsiveness (lower EC_50_: P(f < m) = 0.83), although the 90% credible interval for the log_10_(EC_50_) contrast overlapped zero [−0.43, 0.12]. For 2-phenylethanol, females showed a directional tendency towards steeper dose–response curves than males (Hill slope *n*; Δ*n* = 0.23 [−0.02, 0.54]; P(f > m) = 0.94), although the 90% credible interval for this contrast narrowly included zero. Credible intervals for all other parameter contrasts overlapped zero.

(*E*)-β-farnesene and 2-phenylethanol showed suggestive evidence of sex-related differences in peripheral sensitivity. For (*E*)-β-farnesene, females showed lower posterior median EC_50_ than males (45 vs. 68 µg mL^−1^), with directional evidence of higher female sensitivity (P(f < m) = 0.83; Figure 2). The 90% credible interval for the log_10_(EC_50_) contrast overlapped zero [−0.43, 0.12], indicating that this pattern should be interpreted as directional rather than definitive. For 2-phenylethanol, the Hill slope parameter showed a directional tendency toward a sex-related difference, with females tending to show steeper dose–response curves than males (*n*: 0.97 vs. 0.75; Δ*n* = 0.23 [−0.02, 0.54]; P(f > m) = 0.94), although the 90% credible interval for this contrast narrowly included zero and this pattern should likewise be interpreted as directional rather than definitive.

## 4. Discussion

Multi-method chemical profiling. The chemical ecology of *Leptoglossus chilensis* has not previously been described. To our knowledge, no pheromones, defensive secretions, glandular volatiles, or peripheral olfactory responses have been reported for this species. Therefore, the present study provides an initial chemical and electrophysiological reference for *L. chilensis,* rather than a functional characterisation of its semiochemical system. Because volatile emissions, cuticular compounds, and semi-volatile fractions are captured by different sampling approaches, we used four complementary sampling methods to capture volatile, cuticular, and semi-volatile chemical fractions. DHS and SPME targeted volatile emissions, while CW and SFE accessed cuticular and semi-volatile fractions. The high proportion of method-exclusive compounds in the representative profile (40 of 69 compounds) supports the conclusion that these techniques sampled largely complementary chemicals. This complementarity is also evident in the EAG stimulus panel, with hexanal retained throughout all four methods in the representative profile, while hexyl acetate and hexyl formate were detected in all four methods in the full dataset, but fell below the ≥20% threshold in CW and were therefore retained only in DHS, SPME, and SFE. The detection-frequency pattern in CW further supports analytical consistency rather than instability: a subset of compounds, including an unidentified long-chain hydrocarbon (CW), tetradecane, and undecane, was detected at high and stable frequency in at least one sex, while the source of lower-frequency detections, individual biological variability among separately processed insects versus instrument or analytical variability, cannot be distinguished with the present design.

Chemical profile in a congener context. Several compounds detected in *L. chilensis* overlap with semiochemicals previously reported in congeneric *Leptoglossus* species. In *L. zonatus*, hexanal, 1-hexanol, hexanoic acid, hexyl acetate, and octyl acetate have been characterised as components of the alarm pheromone system [8]. In the present study, hexanal and hexanoic acid were retained across all four representative method profiles, whereas hexyl acetate was detected across all methods in the full dataset and retained (frequency ≥ 20%) in DHS, SPME, and SFE. This overlap suggests that *L. chilensis* shares part of the C_6_ aldehyde/acid/ester chemical background reported from other *Leptoglossus* species [17], although the defensive or alarm function of these compounds in *L. chilensis* remains to be tested directly.

β-caryophyllene was detected exclusively in male DHS collections and was absent from all contact-based extraction methods, including CW. Because CW extracts compounds directly from individual insects without plant material, the absence of β-caryophyllene from this fraction is inconsistent with metathoracic or abdominal glandular production, from which compounds are expected to be recovered by cuticular extraction. Consistent with this, β-caryophyllene was detected in 70% of leaf DHS collections from *D. stramonium* performed under matching conditions (this study), confirming that the host plant itself is a reliable source of this compound in the local headspace. This finding is further supported by previous reports identifying β-caryophyllene as the dominant herbivore-induced sesquiterpene in *Datura wrighti* [30], a congener of the host plant used here, and among herbivore-induced volatiles in cotton attacked by *L. phyllopus* [31]. Collectively, these results indicate that the detection of β-caryophyllene in male insect headspace most likely reflects host plant emission rather than insect-derived production. Male-associated sesquiterpene emissions are documented in *Leptoglossus*, for example, leptotriene in *L. zonatus* and *L. occidentalis* [18] and nerolidol in *L. phyllopus* [32]. Although these are structurally distinct end-products, β-caryophyllene has been identified as the biosynthetic precursor of leptotriene in *L. zonatus* [18]. Leptotriene itself was not detected in any *L. chilensis* extract or extraction method, based on comparison of retention indices and mass spectra with published values. Whether an analogous pathway operates in *L. chilensis* cannot be resolved by the present data, given the strong evidence for plant-derived background levels of β-caryophyllene; targeted analysis under controlled, plant-free dietary conditions would be required to test this possibility directly.

Sex-related differences in chemical profiles. Sex-related differences were more pronounced in the cuticular profile than in volatile emissions. The comparative framework of Aldrich & Yonke [6] established that across Coreoidea, metathoracic gland secretions are chemically similar between sexes and distinctive only at the generic level, while male-specific ventral abdominal gland secretions are species-specific both qualitatively and quantitatively, a division between “defence/alarm” chemistry and “mate/attraction” chemistry at the glandular level. In *L. chilensis*, the strong male bias in long-chain alkanes in CW profiles may reflect sex-specific cuticular hydrocarbon composition, consistent with the well-documented general role of cuticular hydrocarbons as mediators of contact recognition and sexual signalling across insects [33], although this interpretation remains specific to the descriptive CW data reported here and is not directly tested. In the volatile fraction, the exclusive detection of β-sesquiphellandrene in male DHS collections parallels the male-specific sesquiterpene emissions in *L. zonatus* and *L. occidentalis* [18] and the male-specific terpenoids documented in *L. phyllopus* and other nearctic coreids [32], suggesting that male-biased terpene-associated headspace profiles may occur in multiple *Leptoglossus* species, although biosynthetic origin remains unresolved. β-caryophyllene was also detected exclusively in male DHS, but its plant-derived origin cannot be excluded (see above). Conversely, hexanal showed a female bias in both DHS and CW, a pattern whose functional significance remains unclear without behavioural data. The sexual asymmetries in chemical profiles are broadly consistent with the antennal sensilla diversity documented by Taszakowski et al. [34], who identified 14 sensory organ types in *L. zonatus* and *L. occidentalis*, with porous wall sensilla proposed to differ in tuning between host plant volatile detection and pheromone perception. Overall, these sex-associated patterns provide targets for future GC-EAD and behavioural assays, but they should not be interpreted as pheromonal or sex-recognition signals without functional validation.

Peripheral olfactory responses and dose–response modelling. All eight compounds elicited concentration-dependent EAG responses in both sexes, demonstrating peripheral antennal responsiveness across the tested panel. Among the four compounds detected in *L. chilensis* extracts, β-caryophyllene yielded the lowest fitted EC_50_ among the tested compounds under these nominal-concentration delivery conditions (~9 µg mL^−1^) in both sexes; the dose–response curve remained unsaturated (NE†) in males, indicating that *E*_max_ was not estimable within the tested range in that sex, whereas the female curve reached an estimable upper asymptote. Because delivered dose was not measured and volatility differs substantially among the tested compounds, this cannot be interpreted as evidence of greater antennal sensitivity to β-caryophyllene specifically; all EC_50_ comparisons in this study are therefore restricted to within-compound, between-sex contrasts. Consequently, these EC_50_ values reflect the model-fitted inflection parameter rather than an empirically constrained half-maximal concentration, as the upper asymptote was not reached within the tested range. Responsiveness to at least one sesquiterpene hydrocarbon was observed in *L. chilensis*, consistent with reports of sesquiterpene activity in congeners. Millar et al. [18] reported that leptotriene, a male-specific sesquiterpene in *L. zonatus* and *L. occidentalis*, elicited strong EAG responses in both sexes of both species, and field trials showed attraction of both sexes. Our results are consistent with sesquiterpene olfactory sensitivity in *L. chilensis*, though functional interpretation requires behavioural confirmation. Hexanal also showed an unsaturated dose–response curve in females, with EC_50_ remaining unidentifiable (NI) within the tested concentration range in that sex; the male curve reached an estimable *E*_max_ and yielded an identifiable, prior-sensitive EC_50_ estimate. Hexyl acetate and hexyl formate yielded identifiable EC_50_ estimates in the high µg mL^−1^ range (708–3598 µg mL^−1^), with some estimates flagged as prior-sensitive (‡), indicating incomplete constraint by the data.

The four compounds not detected in *L. chilensis* extracts—benzyl alcohol, 2-phenylethanol, (*E*)-β-farnesene, and ocimene—nevertheless elicited clear dose-dependent EAG responses in both sexes. Benzyl alcohol and 2-phenylethanol belong to the aromatic volatile fraction of male-specific 7–8th ventral abdominal gland secretions reported in *Leptoglossus.* Aldrich et al. [23,24] proposed that these male-specific abdominal gland secretions may function as long-range female attractants, although direct behavioural confirmation remains limited. Their detection by *L. chilensis* antennae indicates peripheral responsiveness to this compound class, despite their absence from our extracts. Whether these compounds are produced by *L. chilensis* under conditions not sampled here, or whether this peripheral responsiveness reflects conserved sensitivity without current ecological relevance remains unresolved. Similarly, (*E*)-β-farnesene showed directional evidence of female-biased sensitivity (EC_50_ 45 vs. 68 µg mL^−1^; P(f < m) = 0.83), consistent with GC-EAD responses in *L. zonatus* females to male-produced volatiles [8]. Because the 90% credible interval for the log_10_(EC_50_) contrast overlapped zero [−0.43, 0.12], this pattern should be interpreted as directional evidence rather than a definitive sex-specific difference. This pattern is consistent with, but does not establish, conserved peripheral responsiveness to semiochemical classes reported in congeners. Ocimene, reported as GC-EAD-active in *L. zonatus* [8], elicited dose-dependent responses here, indicating peripheral sensitivity to a compound previously implicated in congener chemical ecology.

Several aspects of the present design warrant caution when interpreting these results. The plant-only DHS control used a longer collection time than the insect protocol (24 h vs. 18 h), which limits direct quantitative comparison between insect and plant headspace concentrations; however, this difference does not affect the qualitative conclusion regarding the presence of β-caryophyllene as a host plant volatile. Three compounds in the representative chemical profile require cautious interpretation: 1,3-dimethylbenzene is likely a laboratory or environmental contaminant rather than an insect-derived compound; 2-heptadecyloxirane may represent an autoxidation artefact of a cuticular hydrocarbon precursor formed during sample processing; and 2,4-dimethylhexane is reported as a tentative identification whose biological origin could not be established. Some EAG parameters should nevertheless be interpreted as phenomenological descriptors of the observed response sequence rather than as definitive estimates of intrinsic antennal sensitivity, and EC_50_ estimates that were sensitive to the prior specification indicate that the tested concentration range did not fully constrain all concentration-response parameters. In addition, the ascending concentration protocol prioritised mapping the response range and detecting low-threshold sensitivity, but did not separate concentration effects from presentation-order effects; future assays should use randomized or blocked stimulus presentation and expanded concentration ranges for compounds showing incomplete or prior-sensitive curves. Behavioural assays will also be required to determine whether the electrophysiological responses reported here correspond to attraction, repellence, alarm, or other behavioural effects. Finally, the biosynthetic origin of β-caryophyllene in *L. chilensis* males remains unresolved; targeted analysis of insects reared on a non-terpenoid artificial diet, combined with isotopic labelling or male-specific gland dissection, would distinguish insect biosynthesis from plant-derived accumulation and clarify whether this compound serves as a precursor in a leptotriene-homologous pathway.

## 5. Conclusions

This study provides the first chemical and electroantennographic reference for *L. chilensis.* Four complementary sampling methods established a representative chemical profile of 69 retained compounds, and all eight EAG stimulus compounds elicited concentration-dependent antennal responses in both sexes, indicating broad peripheral responsiveness across the tested chemical classes. Sex-associated patterns in cuticular and headspace chemistry were statistically supported after multiplicity correction for a subset of compounds; additionally, β-caryophyllene, initially considered a candidate male signal, is more likely to be host plant-derived. These findings provide an initial chemical and sensory basis for future behavioural and monitoring work on this species. This study also contributes to the limited quantitative EAG dose–response literature in Hemiptera: the four-parameter Bayesian hierarchical Hill model used here provides phenomenological descriptors of baseline response (*E*_0_), maximum response (*E*_max_), half-maximal effective concentration (EC_50_), and curve steepness (*n*) [26,27], propagates uncertainty through posterior distributions, and allows incomplete or weakly identified curves to be explicitly flagged. The principal limitations of the present design are that delivered antennal dose was not measured, precluding valid cross-compound EC_50_ ranking; that concentrations were presented in ascending order only, confounding concentration with presentation order at the individual-antenna level; that several curves remained unsaturated or had EC_50_ estimates sensitive to the prior specification; and that retention indices could not be computed for compounds outside the calibrated C_8_–C_28_ range. These limitations define clear priorities for follow-up work rather than undermining the present chemical and electrophysiological reference.

## Figures and Tables

**Table 1:** Detection frequencies by sex in cuticular wash and dynamic headspace profiles.

Method	Detection Pattern	Compound	Female Detection (%)	Male Detection (%)
CW	Higher in males	unidentified long-chain hydrocarbon	10	76
CW	Higher in males	tetradecane	23	86
CW	Higher in males	undecane	20	76
CW	Higher in males	eicosane	17	59
CW	Higher in males	2,4-dimethylhexane ^a^	13	55
CW	Higher in males	heptadecane	23	48
CW	Higher in females	2-heptadecyloxirane ^b^	37	0
CW	Higher in females	hexadecane	33	0
CW	Higher in females	α-pinene	33	0
CW	Higher in females	heptane	27	0
CW	Higher in females	hexanal	63	38
DHS	Higher in males	β-caryophyllene	0	80
DHS	Higher in males	β-sesquiphellandrene	0	50
DHS	Higher in males	2-hexenal	0	50
DHS	Higher in males	hexadecane	0	80
DHS	Higher in males	tetradecane	20	90
DHS	Higher in males	hexanoic acid	10	80
DHS	Higher in females	unidentified long-chain hydrocarbon	60	0
DHS	Higher in females	unidentified long-chain hydrocarbon	60	10
DHS	Higher in females	butyl acetate	30	0
DHS	Higher in females	1,3-dimethylbenzene ^c^	30	0
DHS	Higher in females	eicosane	30	0

Detection frequencies correspond to the percentage of biological replicates in which each compound was detected within each sex and extraction method. This table summarises the compounds discussed in Section 3 as examples of sex-differential detection frequencies. No additional detection frequency threshold, abundance threshold, or formal statistical test was applied for inclusion in this table. CW = cuticular wash; DHS = dynamic headspace sampling. ^a^ Tentative identification; biological origin could not be confirmed (see Section 2). ^b^ Probable autoxidation artefact of a cuticular hydrocarbon precursor (see Section 2). ^c^ Likely laboratory or environmental contaminant (see Section 2). See Table S2 for full identification data across all detected compounds.

**Table 2:** Occurrence and selection rationale of EAG stimulus compounds.

Compound	Rationale for EAG	Basis for Inclusion	Detection in *L. chilensis*
Hexanal	C_6_ aldehyde; alarm/defensive chemistry	[6,8,17]	CW, DHS, SPME, SFE; retained ≥20% in all four methods; detected in both sexes
Hexyl acetate	C_6_ ester; alarm/defensive chemistry	[6,8,17]	CW, DHS, SPME, SFE; retained ≥20% in DHS, SPME, and SFE; detected in both sexes, low-frequency in CW
Hexyl formate	C_6_ ester; class-level analogue within alarm/defensive ester chemistry	C_6_ ester class reported in *Leptoglossus* chemical ecology; authentic standard available	CW, DHS, SPME, SFE; retained ≥20% in DHS, SPME, and SFE; detected in both sexes, low-frequency in CW
β-caryophyllene	Sesquiterpene hydrocarbon; class-level probe for terpene-associated responses	Male-associated sesquiterpene chemistry reported in congeners; authentic standard available	DHS only; retained ≥20% in male DHS; insect-specific production not established
Benzyl alcohol	Aromatic alcohol; male glandular compound reported in *Leptoglossus*	[23,24]	Not detected
2-Phenylethanol	Aromatic alcohol; male glandular compound reported in *Leptoglossus*	[24]	Not detected
(*E*)-β-farnesene	Sesquiterpene hydrocarbon; aggregation-related compound	[8,18]	Not detected
Ocimene	Monoterpene hydrocarbon; GC-EAD-active or behaviourally active compound	[8]	Not detected

Compounds were selected a priori based on semiochemical classes reported in *Leptoglossus* and the availability of authentic standards. Detection in *L. chilensis* was evaluated independently. Detection in *L. chilensis* refers to the full dataset. “Retained ≥ 20%” indicates that the compound met the detection-frequency threshold in at least one sex within the indicated method. Low-frequency detections were present in the full dataset but were not retained in the representative chemical profile. Basis for inclusion indicates either compound-level literature support or class-level rationale within chemical classes reported from *Leptoglossus*.

**Table 3:** EAG model selection by LOO-CV.

Compound	Δelpd	SE Δ	Δelpd/SE Δ	Selected (Obs.-Level LOO; Supplementary)
β-caryophyllene	−40.63	10.40	−3.91	G2
benzyl alcohol	−20.89	13.75	−1.52	G1
(*E*)-β-farnesene	−97.44	15.71	−6.20	G2
hexanal	−29.19	11.55	−2.53	G2
hexyl acetate	−13.78	10.00	−1.38	G1
hexyl formate	−44.91	12.32	−3.65	G2
ocimene	−15.57	11.96	−1.30	G1
2-phenylethanol	−103.18	21.02	−4.91	G2

G1: random intercept on log *E*_0_ only. G2: random intercepts on both log *E*_0_ and log *E*_max_. Δelpd is the difference in expected log pointwise predictive density (elpd), estimated by leave-one-out cross-validation at the observation level (a supplementary diagnostic; see Section 2), calculated as elpd_G1 − elpd_G2; therefore, negative values indicate higher expected predictive performance of G2. SE Δ is the standard error of Δelpd. G2 was selected when Δelpd/SE Δ < −2; otherwise, the simpler G1 model was retained. Absolute per-model elpd and SE values are not tabulated here; only the difference-based diagnostic used for model selection is reported. Model selection using 10-fold cross-validation grouped by antenna—the primary criterion (Section 2)—selected G2 for all eight compounds: β-caryophyllene (Δelpd/SE Δ = −4.62), benzyl alcohol (−3.21), (*E*)-β-farnesene (−7.05), hexanal (−4.92), hexyl acetate (−3.55), hexyl formate (−5.16), ocimene (−3.19), and 2-phenylethanol (−5.76). For benzyl alcohol, hexyl acetate, and ocimene, this differs from the supplementary observation-level comparison in Table 3, which favoured G1; because grouped cross-validation is not subject to the exchangeability limitation of observation-level LOO-CV (Section 2), G2 is reported for all eight compounds in Table 4 and throughout Section 3.

**Table 4:** EAG dose–response model parameters by sex.

Compound	Sex	*E*_0_ [mV]	*E*_max_ [mV]	EC_50_ [µg mL^−1^]	*n*
β-caryophyllene	Female	0.33 [0.29, 0.38]	0.44 [0.40, 0.50]	9 [5, 16]	2.14 [1.21, 4.18]
	Male	0.48 [0.42, 0.55]	NE†	9 § [5, 15]	2.11 [1.20, 4.23]
benzyl alcohol	Female	0.47 [0.40, 0.54]	2.04 [1.61, 2.83]	2624 [1473, 6783]	1.62 [1.03, 3.75]
	Male	0.48 [0.41, 0.55]	1.58 [1.28, 2.07]	2413 [1515, 5784]	2.17 [1.28, 4.39]
(*E*)-β-farnesene	Female	0.33 [0.28, 0.38]	0.61 [0.52, 0.73]	45 [29, 71]	1.17 [0.81, 1.81]
	Male	0.29 [0.24, 0.34]	0.59 [0.50, 0.71]	68 [41, 97]	1.18 [0.80, 2.46]
hexanal	Female	0.35 [0.28, 0.42]	NE†	NI	1.74 [1.15, 3.92]
	Male	0.38 [0.30, 0.47]	0.76 [0.56, 1.22]	3598 ‡ [1478, 18243]	1.49 [0.85, 3.77]
hexyl acetate	Female	0.40 [0.35, 0.46]	0.89 [0.78, 1.06]	708 [373, 1632]	0.83 [0.55, 1.26]
	Male	0.36 [0.31, 0.42]	1.16 [0.97, 1.44]	1596 [700, 5097]	0.59 [0.44, 0.80]
hexyl formate	Female	0.39 [0.34, 0.44]	0.97 [0.80, 1.30]	2411 [1454, 7202]	1.86 [1.07, 4.08]
	Male	0.27 [0.24, 0.31]	0.65 [0.53, 0.99]	3017 ‡ [1512, 12071]	1.85 [1.08, 4.07]
ocimene	Female	0.36 [0.31, 0.42]	1.03 [0.84, 1.34]	2649 ‡ [964, 9202]	0.58 [0.45, 0.79]
	Male	0.36 [0.31, 0.42]	0.81 [0.68, 0.99]	877 ‡ [260, 3557]	0.51 [0.37, 0.71]
2-phenylethanol	Female	0.43 [0.34, 0.53]	2.35 [1.85, 3.07]	3492 [2193, 6952]	0.97 [0.80, 1.26]
	Male	0.50 [0.40, 0.61]	1.90 [1.52, 2.47]	2466 [1434, 6172]	0.75 [0.61, 0.92]

Values are posterior medians with 90% credible intervals. NE† indicates that the dose–response curve did not reach an estimable upper asymptote within the tested concentration range. NI indicates that EC_50_ was not identifiable from the fitted curve. ‡ indicates prior-sensitive EC_50_ estimates where the ratio of posterior medians between the loosest (SD = 2.0) and tightest (SD = 1.0) EC_50_ prior specifications exceeded 2.0. § EC_50_ has a defined posterior but *E*_max_ was not estimable within the tested range (NE†); this estimate reflects the model-fitted inflection parameter rather than an empirically constrained half-maximal concentration.

## Data Availability

The datasets generated and analysed during the current study are available in Zenodo (https://doi.org/10.5281/zenodo.22136329).

## Supplementary Materials

The following supporting information can be downloaded at: https://www.mdpi.com/article/10.3390/insects17090923/s1, File S1: EAG quality-control summary by compound, sex, and recording session; Table S1: Diagnostic flags for EAG dose–response model parameters by compound and sex; Table S2: Compound identification reference for all four extraction methods, including linear retention indices and tentative-identification flags; Figure S1: EAG quality control, drift correction, and Bayesian dose–response model specification; Table S3: Fisher’s exact tests (Benjamini–Hochberg-corrected) for sex differences in detection frequency, all 210 compound × method comparisons.
