# Supplementary material for: Volatile and Cuticular Chemical Profiling and Antennal Dose–Responses in Leptoglossus chilensis (Hemiptera: Coreidae)
Source: Insects. 2026 Sep 3;17(9):923. doi: 10.3390/insects17090923 (PMC13607720; doi:10.3390/insects17090923)
Supplement: Supplementary file 1 [file insects-17-00923-s001.zip › Figure S1 EAG quality control, drift correction, and Bayesian dose–response model specification.pdf]

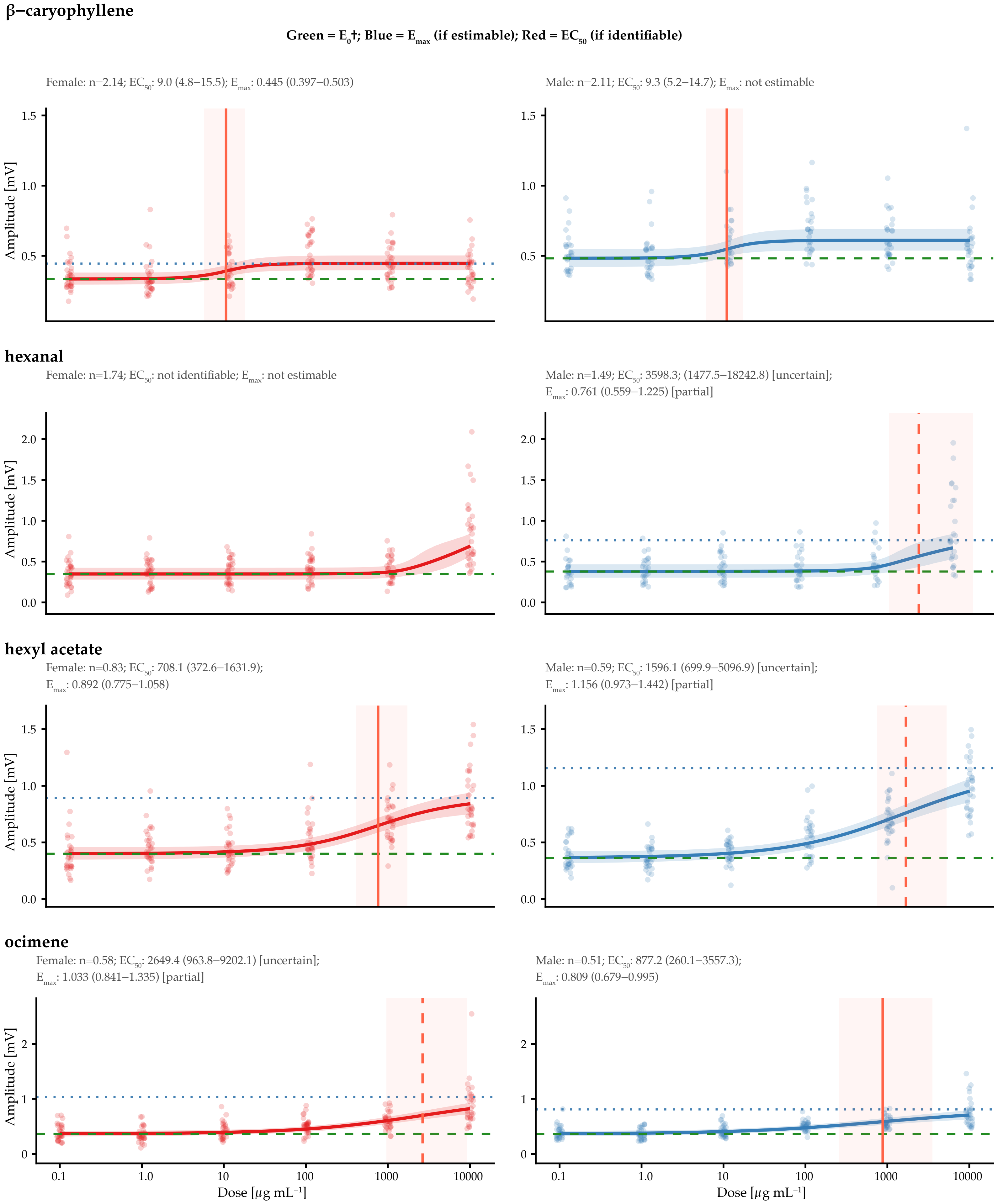

EC_{50} and E_{max} on each panel are posterior median (90% credible interval), shown only when estimable; E_0, n, and P(n>=1) are reported in Table 4 and Table S1.
E0 is the lower asymptote conditional on the minimum tested dose, not the true physiological baseline.
