# Supplementary material for: Volatile and Cuticular Chemical Profiling and Antennal Dose–Responses in Leptoglossus chilensis (Hemiptera: Coreidae)
Source: Insects. 2026 Sep 3;17(9):923. doi: 10.3390/insects17090923 (PMC13607720; doi:10.3390/insects17090923)
Supplement: Supplementary file 1 [file insects-17-00923-s001.zip › File_S1.pdf]

Online Resource 1 — EAG quality control and drift correction procedures

File S1. EAG quality-control summary by compound, sex, and recording session

Per-antenna residual diagnostics from the corrected, post-bugfix Bayesian dose-response model fits (grouped-antenna model, primary selection). MAR = mean absolute residual across all doses for that antenna; max res = maximum absolute residual; n obs = number of observations (doses × replicates) contributing to the antenna. qc_resid flags antennas whose MAR exceeds twice the median MAR across all antennas of that compound and sex.

Compound	Antenna id	Sex	MAR	Max res	n obs / QC flag
β-caryophyllene	female_EAD 1	female	0.066	0.219	18 / ✓
β-caryophyllene	female_EAD 10	female	0.039	0.097	18 / ✓
β-caryophyllene	female_EAD 2	female	0.043	0.135	18 / ✓
β-caryophyllene	female_EAD 3	female	0.036	0.079	18 / ✓
β-caryophyllene	female_EAD 4	female	0.059	0.200	18 / ✓
β-caryophyllene	female_EAD 5	female	0.105	0.331	18 / ✓
β-caryophyllene	female_EAD 6	female	0.061	0.173	18 / ✓
β-caryophyllene	female_EAD 7	female	0.033	0.088	18 / ✓
β-caryophyllene	female_EAD 8	female	0.091	0.237	18 / ✓
β-caryophyllene	female_EAD 9	female	0.078	0.361	18 / ✓
β-caryophyllene	male_EAD 1	male	0.252	0.786	18 / MAR> 2× median
β-caryophyllene	male_EAD 10	male	0.106	0.292	18 / ✓
β-caryophyllene	male_EAD 2	male	0.117	0.313	18 / ✓
β-caryophyllene	male_EAD 3	male	0.045	0.186	18 / ✓
β-caryophyllene	male_EAD 5	male	0.035	0.096	18 / ✓
β-caryophyllene	male_EAD 6	male	0.067	0.218	18 / ✓
β-caryophyllene	male_EAD 7	male	0.047	0.148	18 / ✓
β-caryophyllene	male_EAD 8	male	0.068	0.198	18 / ✓
β-caryophyllene	male_EAD 9	male	0.074	0.156	18 / ✓
benzyl alcohol	female_EAD 1	female	0.070	0.209	18 / ✓
benzyl alcohol	female_EAD 10	female	0.159	0.644	18 / ✓
benzyl alcohol	female_EAD 2	female	0.111	0.380	18 / ✓
benzyl alcohol	female_EAD 3	female	0.164	0.938	18 / ✓
benzyl alcohol	female_EAD 6	female	0.097	0.461	18 / ✓
benzyl alcohol	female_EAD 7	female	0.057	0.134	18 / ✓
benzyl alcohol	female_EAD 8	female	0.140	0.594	18 / ✓
benzyl alcohol	female_EAD 9	female	0.119	0.294	18 / ✓
benzyl alcohol	male_EAD 1	male	0.085	0.391	18 / ✓
benzyl alcohol	male_EAD 10	male	0.062	0.323	18 / ✓
benzyl alcohol	male_EAD 2	male	0.041	0.136	18 / ✓
benzyl alcohol	male_EAD 3	male	0.087	0.426	18 / ✓
benzyl alcohol	male_EAD 4	male	0.058	0.157	18 / ✓
benzyl alcohol	male_EAD 5	male	0.161	0.679	18 / ✓
benzyl alcohol	male_EAD 6	male	0.101	0.689	18 / ✓
benzyl alcohol	male_EAD 9	male	0.168	0.532	18 / ✓
(E)-β-farnesene	female_EAD 1	female	0.114	0.262	18 / ✓
(E)-β-farnesene	female_EAD 10	female	0.065	0.143	18 / ✓
(E)-β-farnesene	female_EAD 2	female	0.052	0.146	18 / ✓
(E)-β-farnesene	female_EAD 3	female	0.067	0.177	18 / ✓
(E)-β-farnesene	female_EAD 4	female	0.053	0.114	18 / ✓
(E)-β-farnesene	female_EAD 5	female	0.087	0.201	18 / ✓
(E)-β-farnesene	female_EAD 6	female	0.066	0.306	18 / ✓

Compound	Antenna id	Sex	MAR	Max res	n obs / QC flag
(E)-β-farnesene	female_EAD 7	female	0.039	0.087	18 / ✓
(E)-β-farnesene	female_EAD 8	female	0.073	0.209	18 / ✓
(E)-β-farnesene	female_EAD 9	female	0.033	0.089	18 / ✓
(E)-β-farnesene	male_EAD 1	male	0.045	0.142	18 / ✓
(E)-β-farnesene	male_EAD 10	male	0.100	0.578	18 / ✓
(E)-β-farnesene	male_EAD 2	male	0.110	0.379	18 / ✓
(E)-β-farnesene	male_EAD 3	male	0.055	0.125	18 / ✓
(E)-β-farnesene	male_EAD 4	male	0.019	0.060	18 / ✓
(E)-β-farnesene	male_EAD 5	male	0.095	0.229	18 / ✓
(E)-β-farnesene	male_EAD 7	male	0.064	0.132	18 / ✓
(E)-β-farnesene	male_EAD 8	male	0.072	0.229	18 / ✓
(E)-β-farnesene	male_EAD 9	male	0.063	0.218	18 / ✓
hexanal	female_EAD 1	female	0.083	0.609	18 / ✓
hexanal	female_EAD 10	female	0.116	1.043	18 / ✓
hexanal	female_EAD 2	female	0.096	0.317	18 / ✓
hexanal	female_EAD 3	female	0.056	0.286	18 / ✓
hexanal	female_EAD 4	female	0.081	0.489	18 / ✓
hexanal	female_EAD 5	female	0.028	0.117	18 / ✓
hexanal	female_EAD 6	female	0.132	0.544	18 / ✓
hexanal	female_EAD 7	female	0.029	0.120	18 / ✓
hexanal	female_EAD 8	female	0.058	0.216	18 / ✓
hexanal	female_EAD 9	female	0.114	0.807	18 / ✓
hexanal	male_EAD 1	male	0.067	0.458	18 / ✓
hexanal	male_EAD 2	male	0.064	0.154	18 / ✓
hexanal	male_EAD 3	male	0.131	1.004	18 / ✓
hexanal	male_EAD 4	male	0.106	0.520	18 / ✓
hexanal	male_EAD 5	male	0.061	0.614	18 / ✓
hexanal	male_EAD 6	male	0.065	0.639	18 / ✓
hexanal	male_EAD 7	male	0.110	0.521	18 / ✓
hexanal	male_EAD 9	male	0.052	0.287	18 / ✓
hexyl acetate	female_EAD 1	female	0.214	0.550	18 / MAR > 2× median
hexyl acetate	female_EAD 10	female	0.081	0.240	18 / ✓
hexyl acetate	female_EAD 2	female	0.063	0.245	18 / ✓
hexyl acetate	female_EAD 3	female	0.045	0.207	18 / ✓
hexyl acetate	female_EAD 4	female	0.079	0.370	18 / ✓
hexyl acetate	female_EAD 5	female	0.060	0.171	18 / ✓
hexyl acetate	female_EAD 6	female	0.090	0.272	18 / ✓
hexyl acetate	female_EAD 7	female	0.113	0.445	18 / ✓
hexyl acetate	female_EAD 8	female	0.090	0.381	18 / ✓
hexyl acetate	female_EAD 9	female	0.125	0.675	18 / ✓
hexyl acetate	male_EAD 1	male	0.133	0.513	18 / ✓
hexyl acetate	male_EAD 10	male	0.099	0.499	18 / ✓
hexyl acetate	male_EAD 2	male	0.106	0.272	18 / ✓
hexyl acetate	male_EAD 3	male	0.104	0.259	18 / ✓
hexyl acetate	male_EAD 4	male	0.096	0.213	18 / ✓
hexyl acetate	male_EAD 5	male	0.097	0.234	18 / ✓
hexyl acetate	male_EAD 6	male	0.095	0.275	18 / ✓
hexyl acetate	male_EAD 7	male	0.105	0.404	18 / ✓
hexyl acetate	male_EAD 8	male	0.092	0.454	18 / ✓
hexyl acetate	male_EAD 9	male	0.090	0.285	18 / ✓
hexyl formate	female_EAD 1	female	0.080	0.321	18 / ✓

Compound	Antenna id	Sex	MAR	Max res	n obs / QC flag
hexyl formate	female_EAD 10	female	0.058	0.212	18 / ✓
hexyl formate	female_EAD 2	female	0.081	0.316	18 / ✓
hexyl formate	female_EAD 3	female	0.062	0.137	18 / ✓
hexyl formate	female_EAD 4	female	0.082	0.295	18 / ✓
hexyl formate	female_EAD 5	female	0.064	0.201	18 / ✓
hexyl formate	female_EAD 6	female	0.067	0.211	18 / ✓
hexyl formate	female_EAD 7	female	0.044	0.091	18 / ✓
hexyl formate	female_EAD 8	female	0.060	0.144	18 / ✓
hexyl formate	female_EAD 9	female	0.163	0.882	18 / MAR > 2× median
hexyl formate	male_EAD 1	male	0.072	0.196	18 / ✓
hexyl formate	male_EAD 10	male	0.033	0.088	18 / ✓
hexyl formate	male_EAD 2	male	0.091	0.509	18 / ✓
hexyl formate	male_EAD 3	male	0.058	0.222	18 / ✓
hexyl formate	male_EAD 4	male	0.113	0.303	18 / ✓
hexyl formate	male_EAD 5	male	0.049	0.182	18 / ✓
hexyl formate	male_EAD 6	male	0.074	0.241	18 / ✓
hexyl formate	male_EAD 7	male	0.058	0.133	18 / ✓
hexyl formate	male_EAD 8	male	0.056	0.120	18 / ✓
hexyl formate	male_EAD 9	male	0.110	0.459	18 / ✓
ocimene	female_EAD 1	female	0.125	0.326	18 / ✓
ocimene	female_EAD 10	female	0.093	0.267	18 / ✓
ocimene	female_EAD 2	female	0.063	0.156	18 / ✓
ocimene	female_EAD 3	female	0.114	0.263	18 / ✓
ocimene	female_EAD 4	female	0.057	0.163	18 / ✓
ocimene	female_EAD 5	female	0.066	0.169	18 / ✓
ocimene	female_EAD 6	female	0.106	0.275	18 / ✓
ocimene	female_EAD 7	female	0.061	0.258	18 / ✓
ocimene	female_EAD 8	female	0.075	0.156	18 / ✓
ocimene	female_EAD 9	female	0.241	1.534	18 / MAR > 2× median
ocimene	male_EAD 1	male	0.074	0.498	18 / ✓
ocimene	male_EAD 10	male	0.067	0.140	18 / ✓
ocimene	male_EAD 2	male	0.089	0.166	18 / ✓
ocimene	male_EAD 3	male	0.062	0.158	18 / ✓
ocimene	male_EAD 4	male	0.061	0.138	18 / ✓
ocimene	male_EAD 5	male	0.045	0.159	18 / ✓
ocimene	male_EAD 6	male	0.045	0.105	18 / ✓
ocimene	male_EAD 7	male	0.091	0.386	18 / ✓
ocimene	male_EAD 8	male	0.053	0.145	18 / ✓
ocimene	male_EAD 9	male	0.095	0.416	18 / ✓
2-phenylethanol	female_EAD 1	female	0.071	0.314	18 / ✓
2-phenylethanol	female_EAD 10	female	0.130	0.702	18 / ✓
2-phenylethanol	female_EAD 2	female	0.089	0.356	18 / ✓
2-phenylethanol	female_EAD 3	female	0.120	0.596	18 / ✓
2-phenylethanol	female_EAD 4	female	0.067	0.326	18 / ✓
2-phenylethanol	female_EAD 5	female	0.096	0.337	18 / ✓
2-phenylethanol	female_EAD 7	female	0.059	0.194	18 / ✓
2-phenylethanol	female_EAD 8	female	0.056	0.147	18 / ✓
2-phenylethanol	female_EAD 9	female	0.063	0.268	18 / ✓
2-phenylethanol	male_EAD 1	male	0.095	0.411	18 / ✓
2-phenylethanol	male_EAD 10	male	0.059	0.219	18 / ✓
2-phenylethanol	male_EAD 2	male	0.058	0.299	18 / ✓

Compound	Antenna id	Sex	MAR	Max res	n obs / QC flag
2-phenylethanol	male_EAD 3	male	0.177	0.675	18 / ✓
2-phenylethanol	male_EAD 4	male	0.151	0.459	18 / ✓
2-phenylethanol	male_EAD 5	male	0.130	0.849	18 / ✓
2-phenylethanol	male_EAD 6	male	0.103	0.590	18 / ✓
2-phenylethanol	male_EAD 7	male	0.074	0.217	18 / ✓
2-phenylethanol	male_EAD 8	male	0.116	0.738	18 / ✓
2-phenylethanol	male_EAD 9	male	0.110	0.369	18 / ✓

S2. Linalool reference and drift correction

Raw EAG amplitudes were corrected for electrode drift using repeated presentations of a reference stimulus (linalool, 1000 μg mL^{-1}) as within-session anchors. Each response was multiplied by the ratio of the first anchor amplitude to the amplitude of the nearest preceding anchor, normalising all measurements to the initial electrode sensitivity. Anchor ratios were computed separately for each antenna and sex.

Antennas were excluded from analysis if the response to a secondary reference stimulus (linalool, 10,000 μg mL^{-1}) declined by more than 40% between the first and last presentation, indicating excessive electrode fatigue. This fatigue-QC filter was applied per antenna and sex. Solvent (hexane) responses were retained for diagnostic inspection but excluded from dose-response modelling; the lower asymptote E0 therefore represents the fitted function at the minimum tested concentration (0.1 ppm), not the true resting baseline.

S3. Bayesian four-parameter Hill model

Dose-response model

Dose-response relationships were described using a four-parameter Hill function, where E0 is the lower asymptote, Emax the upper asymptote, EC_{50} the dose producing 50% of the maximal response, and n the Hill coefficient governing response steepness. All four parameters were constrained to be positive via log-transformation. This functional form was assumed based on standard dose-response convention and was not compared against alternative functional forms. Posterior predictive checks were used to evaluate overall fit and residual behaviour.

Models were fitted within a Bayesian hierarchical framework using brms with CmdStan as the backend. Individual antennas were treated as the sampling unit. Two random-effects structures were compared. In the baseline model (G1), log(E0) was modelled with sex-specific population means and a random intercept per antenna, whereas log(Emax), log(EC50), and log(n) were modelled as sex-specific fixed effects. In the extended model (G2), both log(E0) and log(Emax) included random intercepts per antenna, while log(EC50) and log(n) remained sex-specific fixed effects. Thus, G1 allowed between-antenna variation in baseline response only, whereas G2 allowed between-antenna variation in both baseline and maximum response. In both G1 and G2, random-effect standard deviations were estimated as common across sexes for each random-effect parameter. This implies equal between-antenna variability in females and males, an assumption imposed by the current model structure.

Observation-level errors followed a Student-t distribution with estimated degrees of freedom ν. EAG amplitude is an aggregate electrical field signal reflecting the summed activity of olfactory receptor neuron populations; between-individual differences in receptor density and physiological state can produce heavy-tailed variation that a Gaussian distribution may not accommodate. The Student-t family was therefore specified a priori as a robust error distribution

for this signal type. Low estimated ν values ($\nu < 5$) indicate that the model required heavier tails than a Gaussian to describe the observed residual variation. This does not indicate model mis-specification by itself, but rather supports the use of the Student-t observation model.

Model selection used leave-one-out cross-validation (LOO-CV; Vehtari et al. 2017), implemented at the observation level. Because LOO-CV conditions on the remaining observations from the same antenna when predicting a held-out observation, absolute elpd values may overestimate predictive performance for new antennas. LOO-CV was therefore used only for relative comparison between G1 and G2. Δelpd was defined as elpd_G1 − elpd_G2; therefore, negative values favour G2. G2 was retained when $\Delta\text{elpd}/\text{SE}\ \Delta < -2$; otherwise, G1 was retained for parsimony. For the manuscript's primary model-selection criterion, this comparison was instead performed with grouped-antenna (10-fold) cross-validation, which respects the non-independence of repeated measurements on the same antenna; the observation-level comparison above is retained here as a supplementary diagnostic (see manuscript Table 3).

Priors

Weakly informative priors were specified on the log scale: $\log(E_0) \sim \text{Normal}(\log(0.35), 0.4)$, $\log(E_{max}) \sim \text{Normal}(\log(1.0), 0.5)$, $\log(EC_{50}) \sim \text{Normal}(\log(100), 1.5)$, $\log(n) \sim \text{Normal}(\log(2), 0.5)$, $\sigma \sim \text{Exponential}(2)$, $\nu \sim \text{Gamma}(2, 0.1)$. The prior on $\log(EC_{50})$ spans approximately four orders of magnitude (5th–95th percentile: ~0.4–22,000 ppm), placing minimal constraint on EC_{50} relative to the experimental range. Prior sensitivity was assessed by refitting the selected model with narrower (sd = 1.0) and broader (sd = 2.0) priors on $\log(EC_{50})$. For compounds where EC_{50} was not identifiable within the experimental range, the parameters most influenced by prior specification are $\log(E_{max})$ and $\log(n)$; the sensitivity analysis does not fully quantify prior dependence for those compounds.

All models were run with 4 chains × 2,000 post-warmup draws (adapt_delta = 0.99). Convergence was assessed via R-hat < 1.01 and bulk ESS > 400 for all fixed-effect parameters.

Influence of atypical antennas

Prior to dose-response fitting, individual antenna profiles were inspected visually. Following model fitting, a per-antenna residual audit was conducted: the mean absolute residual (MAR) was computed for each antenna across all doses, and antennas with MAR exceeding twice the median MAR across all antennas were flagged (see S1 above). These antennas passed the fatigue quality control threshold but showed dose-response behaviour inconsistent with the remainder of the sample. We note that with n = 10 antennas per sex, this threshold identifies deviation from the current sample, not from the population distribution of peripheral sensitivity, which is unknown for this species.

For compounds with flagged antennas, the selected model was refit on the reduced dataset with flagged antennas excluded. For each parameter and sex, robustness was assessed as the percentage shift in posterior median between full and reduced fits, and whether 90% credible intervals overlapped. Parameters showing less than 20% shift and overlapping intervals were considered robust to the exclusion. Where robustness was confirmed, full-dataset results are reported as primary; where parameters were sensitive to exclusion, this is declared explicitly.

Estimand reporting

EC50 was considered identifiable if ≥ 80% of posterior draws fell within the experimental dose range [0.1–10,000 ppm]. EC_{50} estimates where 80–100% of draws fell within the range but the 90% credible interval extended beyond half the maximum tested dose are reported as identifiable with

high uncertainty. When fewer than 80% of draws were within range, EC_{50} is reported as not identifiable within the experimental range. Emax is reported as not estimable when the curve did not saturate within the tested range, as its posterior is then dominated by the prior rather than the data. When the curve did not saturate, that is when EC_{50} was not identifiable, the Hill coefficient n and the derived maximum slope are likewise prior-influenced and are flagged accordingly. $P(n \geq 1)$, the posterior probability that the dose-response is sigmoidal or steeper, is reported as a descriptive quantity but interpreted with caution under these conditions, as it reflects the prior on log(n) more than the data. Curve shape was not classified categorically; any threshold-based classification would constitute an analytical decision without specific empirical support for this species.

Sex differences in E_0, EC_{50}, n, and maximum slope were quantified as posterior contrasts (female − male) computed draw-by-draw, propagating full parameter uncertainty. The EC_{50} contrast was computed on the log10 scale and reported as a ratio. The Emax contrast was not computed for compounds where Emax was not estimable in one or both sexes. Contrasts for n and slope were not computed for compounds where n was prior-influenced in both sexes, as a credible difference between sexes in a prior-dominated parameter does not constitute interpretable biological evidence.

Results describe peripheral olfactory sensitivity as measured by electroantennography. EAG amplitude is an aggregate electrical signal that cannot be unambiguously attributed to the compound of interest independently of solvent or reference-stimulus interactions. Replicate measurements within each antenna were treated as conditionally independent given the random effect; temporal order within session was not modelled. Behavioural relevance of the reported sensitivities requires independent validation.
